# Financial vulnerability, health outcomes, and well-being of older adults during the COVID-19 pandemic

**DOI:** 10.7189/jogh.12.03021

**Published:** 2022-04-23

**Authors:** Shadrack Osei Frimpong, Francis Arthur-Holmes, Williams Agyemang-Duah

**Affiliations:** 1 Department of Public Health and Primary Care, University of Cambridge, Cambridge, UK; 2 Cocoa360, Accra, Ghana; 3 Department of Sociology and Social Policy, Lingnan University, Tuen Mun, Hong Kong; 4 Department of Geography and Planning, Queen’s University, Kingston, Ontario, Canada

The 2019 novel coronavirus (COVID-19), which started in Wuhan in China, revealed the fragility of many health systems. In developing countries like Ghana, vulnerable populations such as older adults have been exposed to health, social, and financial risks. Existing evidence shows that older adults are at a higher risk of contracting the disease due to their declining immune functioning and challenges with comorbid health conditions [1]. Consequently, older populations require protection and support to manage their living conditions during the COVID-19 pandemic.

Several studies and commentaries have highlighted the negative consequences of the COVID-19 pandemic on older adults’ health concerns, including increased risk of elder abuse and neglect [2,3] and social isolation leading to higher risks of cardiovascular disease, neurocognitive decline, and mental health problems [4]. The fear of COVID-19 infection has created emotional insecurity and anxiety disorders. These physical and emotional challenges make older populations more susceptible to financial difficulties during the pandemic [4]. Protecting older adults from exposure to the virus remains an essential component of maintaining their health and well-being during the global pandemic.

While much has been written on the impact of the COVID-19 pandemic, there is a paucity of evidence on the linkage between the financial vulnerability caused by the COVID-19 pandemic and health outcomes of older adults aged 60+ in sub-Saharan Africa (SSA). Investigating the economic impact of the COVID-19 pandemic and its effect on older adults’ health outcomes and well-being is crucial for developing social interventions that seek to provide financial support to vulnerable older populations so that their health does not deteriorate. In this viewpoint, we highlight some dynamics of older adults aged 60+ and their financial vulnerability and health outcomes during the global COVID-19 crisis, which has not been studied so far.

**Figure:**
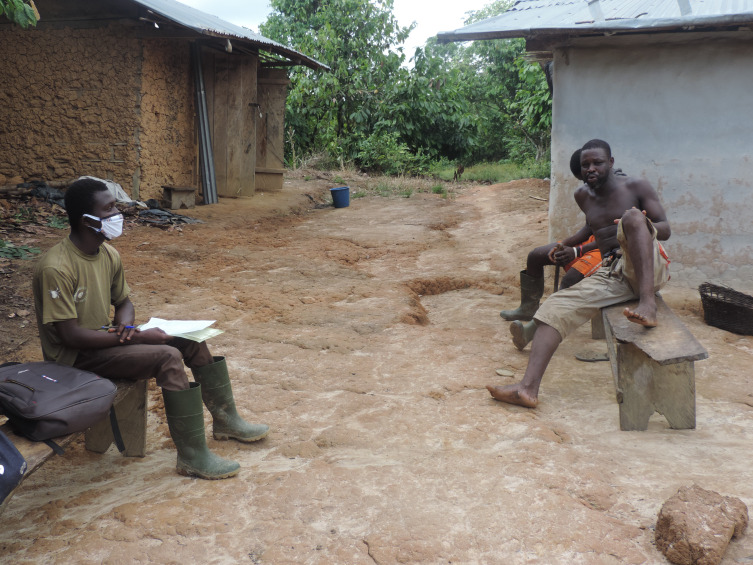
Photo: Older adults in Ghana receiving COVID-19 public health education. Source: Communications Team, Cocoa360 (used with permission).

## COVID-19, FINANCIAL IMPACT, AND OLDER ADULTS IN SUB-SAHARAN AFRICA

Older populations, especially those aged 60+ in SSA, have faced financial challenges during the pandemic. The global crisis has affected household incomes, especially those of citizens of low- and middle-income countries. However, older Africans may face different financial impacts because of ageing demographics and economic status. For instance, older adults aged 50-59 may still receive salaries from their work in the formal sector compared to those whose jobs are at risk in the informal sector during the COVID-19 pandemic. Besides, older adults aged 60+ have a higher likelihood of experiencing the financial impact of the COVID-19 pandemic because most of them are on retirement and depend on their pension package and social networks for survival.

While older adults in the informal sector will not be affected by retirement age due to a lack of regulations for their work, they may stop working due to health challenges and concerns, including their exposure to the virus. In the context of Ghana, Arthur-Holmes and Agyemang-Duah [5] reported that, due to the financial impact of the COVID-19 pandemic, older adults’ support services and income inflow will be affected when their children lose their source of livelihood. This subsequently increases older adults’ vulnerability and affects their access to social services.

Like the active working population, older adults aged 60+ who do not have any occupation are more likely to suffer from the financial impact of the COVID-19 crisis. This might happen in different ways. For example, the pandemic causes people to lose their jobs and consequently affects their capacity to provide for their older parents or relatives who depend on them for financial assistance. Lee et al. [6] mentioned that older adults who are self-employed or not in white-collar jobs are significantly more likely to report financial problems during the COVID-19 pandemic.

Also, unemployed older groups who rely on non-profit organisations for financial support may not receive it during the COVID-19 pandemic, as some organisations’ finances are primarily impacted. Older adults cannot work during the COVID-19 pandemic because of their underlying health conditions and will struggle to maintain their income inflow and take care of their essential needs [6].

In addition, government COVID-19 response measures such as lockdowns and social distancing impact informal older workers’ finances, mainly when they are restricted from selling in the markets or on the streets. For instance, during the three-week partial lockdown in Ghana, some older Ghanaians who trade in the market areas suffered financially due to market closures and reduced sales. These are outcomes of social distancing measures during the pandemic [5].

## OLDER ADULTS AND HEALTH OUTCOMES OF THE FINANCIAL IMPACTS OF COVID-19 PANDEMIC IN SUB-SAHARAN AFRICA

Evidence from literature shows that older people are inflicted with non-communicable and communicable diseases and other health problems, including cognitive impairment, hypertension, diabetes, anaemia, chronic respiratory diseases, cancers, ischemic heart diseases, and kidney issues [7]. These multiple health conditions significantly impact their health outcomes [8]. For older adults who encounter financial challenges, their health outcomes will be further exacerbated. These adverse health outcomes may manifest in various ways.

First, older adults whose source of livelihoods have been negatively impacted may worry about them, resulting in anxiety disorders and other psychological problems. This situation may trigger other health issues that stem from their inability to participate in social and recreational activities such as religious gatherings and wedding ceremonies due to financial challenges. Before the COVID-19 pandemic, more than 20% of older populations worldwide suffered from mental or neurological disorders, including dementia and depression, and 3.8% of older people suffered from an anxiety disorder [9].

Second, considering that chronic non-communicable diseases require regular and special care, older adults who may experience financial difficulties during the COVID-19 pandemic will find it hard to cover their health care expenses to manage their illnesses. Such difficult conditions are more likely to worsen their health conditions and later result in fatalities. Some older adults may die due to a lack of funds for surgical operations and medications. This clearly shows that the older adults’ health care expenditure is likely to be affected by the financial impact of the COVID-19 crisis, as they may not have a constant inflow of cash from their families, friends and other support groups. They are more likely to have a health care cost burdens.

Moreover, older adults are likely to face food insecurity due to financial challenges. Those without sufficient funds cannot purchase enough food items or meals. This challenge may culminate in hunger with severe implications for their physical and mental health and well-being. Older adults, especially those with low-income families, may not consume nutritional food during the pandemic. Generally, studies have shown that food insecurity is associated with lower nutrient intake and adverse health outcomes among older adults [10-12]. This may pose a significant public health concern for older adults in SSA who are experiencing pandemic-related financial problems.

**Figure 1:**
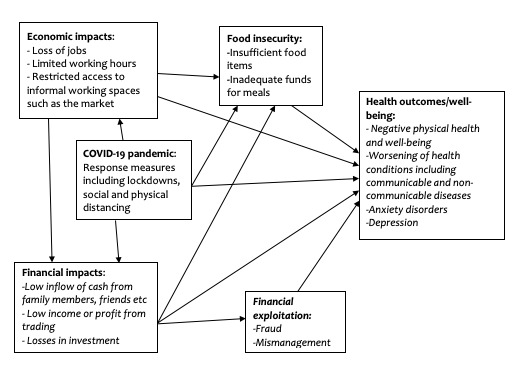
Relationship between the COVID-19 pandemic, its economic/financial effects and health outcomes, and the well-being of older Ghanaian adults aged 60+.

Lastly, the financial impact of the COVID-19 crisis may cause caregivers to exploit older adults in their care. With limited funds, caregivers may attempt to exploit older adults financially, especially when their funds have been spent and they do not have any other means of taking care of themselves. Existing evidence points to the potential existence of such abuses, which occur in the form of money extortion during the COVID-19 pandemic [2]. Such abuses and their consequent stressors may affect older adults’ psychological and mental well-being. Experiencing financial abuse can also make older adults feel like they are not controlling their finances during the pandemic, which could lead to anxiety. We constructed a diagram to illustrate the dynamics of economic/financial impacts of COVID-19 on the health outcomes and well-being of older adults aged 60+ (**Figure 1**).

## LOOKING FORWARD: SOCIAL INTERVENTIONS AND SOCIAL SUPPORT FOR OLDER ADULTS IN SUB-SAHARAN AFRICA

Financial vulnerability threatens older adults’ health and quality of life in impoverished households. It is therefore crucial that policymakers, non-governmental organisations (NGOs), and geriatric institutions in SSA implement policies to meet the needs and improve the well-being of older persons during and after the COVID-19 crisis. Government and civil society, including traditional authorities and NGOs, could help address the financial impact of the pandemic on the health outcomes and well-being of older adults in SSA in a myriad of ways.

First, respective governments of sub-Saharan African countries could design social intervention programmes that identify older adults who have been severely affected by the pandemic and struggle to obtain funds to meet their basic needs. Next, they could provide such vulnerable older adults with financial support to relieve their economic plight. In Ghana, the government already has the Livelihood Empowerment against Poverty (LEAP) programme, which provides bi-monthly funds to beneficiaries’ older adults. Those who are not captured on the LEAP programme but face financial challenges during the pandemic could be added to the beneficiaries’ list. Government cash transfers in various sub-Saharan African countries should be increased to cover more expansive geographical locations and older population groups. Besides, welfare relief funds could purposely be established to help older adults whose sources of financial assistance are damaged by this pandemic.

We also highlight the recommendations from existing literature regarding social security and retirement schemes to reduce the financial impact of the pandemic among older adults. Social security institutions in SSA can make provisions for employed and unemployed older adults who are economically and financially impacted by the pandemic, to receive a portion of the retirement package to meet their basic needs, especially food [5].

Finally, public and private groups such as NGOs and social protection groups also have essential roles in improving the financial conditions of older adults during these critical times. Religious groups and NGOs could identify the vulnerable older population in their organisations and the community in which they are operating and provide them with financial assistance. Such assistance would be crucial in protecting older adults from food insecurity problems and their attendant effects on their health and well-being. Geriatric institutions and social protection groups should organise community sensitisation programmes to call upon families, neighbours, and friends to support older adults financially.

## CONCLUSION

Given the adverse outcomes of the COVID-19 pandemic, the financial impact of the COVID-19 pandemic on the health and well-being of older adults in SSA is an important area for research and practice that needs more attention. Many older adults are at a higher risk of facing food insecurity and poor health outcomes due to their financial struggles during the pandemic. However, this important phenomenon in SSA and other regions remains underexplored in studies at the moment. This challenge also presents an opportunity for researchers to examine the effects of the financial vulnerability caused by the COVID-19 pandemic on health outcomes of older adults, so as to find evidence for informing social interventions during and after the pandemic.
